# Supplementary material for: Examining the prevalence and determinants of early initiation of breastfeeding: Evidence from the 2017/2018 Benin demographic and health survey
Source: PLOS Glob Public Health. 2023 Aug 16;3(8):e0002278. doi: 10.1371/journal.pgph.0002278 (PMC10431659; doi:10.1371/journal.pgph.0002278)
Supplement: Attachment — Submitted filename: RESPONSE TO REVIEWS.docx [file pgph.0002278.s001.docx]

**AUTHORS’ RESPONSE TO REVIEWS**

Title: **Examining the prevalence and determinants of early initiation of breastfeeding: Evidence from the 2018 Benin Demographic and Health Survey**

**Dear Editor and Reviewers,**

We convey our gratitude to you for the critical and constructive review which has subsequently led to some alterations of our paper entitled “**Examining the prevalence and determinants of early initiation of breastfeeding: Evidence from the 2017/2018 Benin demographic and health survey**”. We have revised the manuscript based on the comments raised. In the following detailed response, we address each comment calling for changes point-by-point, indicating where relevant additional texts have been added to the body of the manuscript. Most of the changes have been indicated in yellow colour. We believe the manuscript has been improved substantively and we look forward to its publication in your reputable journal, PLoS Global Public Health

**Version:**1

**Date:** 03/04/2023

Reviewer #1: Review Comments

Thank you for the opportunity to review this paper. Though an important topic, there are several issues the authors should address before the paper can be considered for publication.

Comments/ Suggestions

Abstract
1 Avoid abbreviations in the abstract section

Response: Thank you. This has been addressed

2. Method should be revised and edited?

Response: Thank you. The method section has been addressed accordingly in lines 37-41 to now read ***“****This is a secondary data analysis of the 2017/2018 Benin demographic and health survey. The study included 7,223 women between the ages of 15 and 49. STATA was used for the data analysis. We used a multilevel binary logistic regression to investigate the factors of early breastfeeding initiation in Benin. To determine the significant relationships, the data were reported as odds ratios (ORs) with 95% confidence intervals (CIs) and p-value 0.05****”****.*

3. Conclusion: it could be revised hence you stated recommendations without some presentation of results? Before recommendation you should summarize results and provides suggestions to the concerned body?

Response: Thank you for the observation. We believe this issue has been addressed well. See lines 54-61

Background
1. Grammatical error and some paragraphs are poorly English written. Generally revision should be needed.

Response: Thank you, that has been done

Methods
1. Line 178-180 is confusing rewrite in clear way or you should remove it.

Response: This has been removed

2. What could be your justification to use LLR and AIC for model comparison; is there any conditions to use these interchangeably for model comparison? How?

Response: LLR and AIC was done to compare models in table 2. That is, the LLR test measures how data are observed in a model while the AIC test shows how well a model fits the data that was analyzed.

Result
1 Line 182 presented about the overall prevalence of EIBF i.e. point estimation. You must mention the interval estimation of the prevalence.

Response: Thank you for the observation. The authors believe this has been resolved “From the study, the prevalence of EIBF among mothers was 56% (95% CI=0.54-0.57). See line 202-203

2. How do you measure media exposure?

Response: This has been explained in lines 161-165

3. In the results section you mention model building i.e. measure of variation ICC …. But I did not see anything about that in the methods part. The result should be in line with methods part?

Response: Thank you. This has been addressed in lines 206-211

3. You should mention the mathematical formula how to compute ICC and others?

Response: Thank you. We believe this has been addressed in line 189-194

Discussion
1. Line 288-291 the sentence should be inconvenience should remove it or rewrite in clear cut way.

Response: Thank you. The sentence has been re-written.

2. The justification regarding the prevalence is poorly explain please improve it by accounting the specific reasons found at hand in Benin?

Response: This has been improved. See lines 289-291

3. Line 273-276 this paragraph was implication of the study but the implication of the study should mention the recommendation parts. Be focus on the nationalities of the study and justify it more about your findings with other studies published elsewhere.

Response: Thank you for the comment. This has been addressed

4. Your discussion focusing only USA and Nepal why? You should widening your eye to see the variation and similarity of findings published in Africa and elsewhere? Hence you get full insight if you compare similar country published on it.

Response: Thank you for this comment. However, our study this not use literature from USA. Nonetheless, authors have widened the scope to include significant studies from sub-Saharan Africa.
5. Generally your discussion needs extensive revision hence the current discussion is shallow, you explain in short- and clear-cut way.

Response: Thanks for the observation this has been addressed

Conclusion
1. Line 328-329 not convincing recommendation? The recommendation should be mention in specific and targeted way.

Response: Thank you. We believe this has been addressed accordingly

2. Please minimize your recommendation hence the current one is too long and difficult to understand? As stated before the recommendation should be short, clear and specific to the targeted authority and individual?

Response: Thank you for the comment. The authors have made changes to this section

Reviewer #2: Overview:

The primary purpose of this study was to examine the prevalence and determinants of early initiation of breastfeeding in Benin. This topic is interesting and relevant, given the far-reaching implications early initiation of breastfeeding can have on the health and wellbeing of newborn infants and mothers. However, major revisions are needed to strengthen the ideas in the paper.

Comments
Abstract:

1.  The first sentence in the abstract seems to suggest that early initiation of breastfeeding is beneficial only in low- and middle-income countries and not in other contexts. Is that what the authors are implying? If not, please rephrase the sentence to suggest that regardless of context, early initiation of breastfeeding is crucial for avoiding early childhood fatalities.

Response: Thank you, changes have been made. See lines 28-29

2.  It is not relevant to indicate which version of Stata was used for the analysis in the result section of the abstract.

Response: Changes have been made

3.  Several of the variables listed in the conclusion section of the abstract are not statistically significant and should be excluded.

Response: Thanks for the observation, changes have been made

4. Also, it is not clear what the authors mean by “health professionals must minimize indications of caesarean delivery.”

Response: This has been removed

Introduction:
1. There is no clear connection between the ideas in the first two sentences in the background section of the paper. The authors noted that “Globally, in 2017, only 50% of newborn babies were breastfed within an hour and 60% of them were exclusively breastfed [1]. To improve logical flow, it would be great if the authors could briefly explain why this is the case and link this to discussions around child survival before they highlight Child survival is a major public health concern in low- and middle-income countries.

Response: Thank you for the comment, this “Early initiation of breastfeeding (EIBF) has proven in studies to avert about a quarter of newborn fatalities in the last decade, but delaying initiation of breastfeeding beyond an hour increases the risk of neonatal mortality” has been added. See lines 74-76

2.  On line 82, the authors note that “As of 2016, EIBF had prevented more than twenty-thousand breast cancer-related deaths [11].” I recommend the authors remove this sentence. They cannot make this claim based on a cross-sectional study conducted in rural Niger.
Response: Thank you for the observation, this has been removed

3. They also note that “EIBF has prevented almost a quarter of neonatal deaths in the last decade [1, 11 (see line 84).” Is this globally or in low- to middle-income countries? Kindly present some statistics to support this claim.

Response: Thanks for the comment worldwide has been added to. See lines 74-76.

4. The authors note that “This study seeks to contribute to research related to EIBF in Benin by investigating the prevalence and determinants of EIBF in the country (see line 103).” Which determinants are they focusing on and why? They need to include a review of the key literature that informed the selection of their variables of interest and explain why we should expect them to have an effect on EIBF.

Response: Thank you for the comment. This had been Previous studies have suggested that women’s employment status, educational level, parity, age, mode of delivery, place of delivery, wealth status, place of birth, antenatal care (ANC) visit, place of residence, mass media, community literacy level and community socioeconomic status are associated with EIBF. See lines 101-104

Materials and Methods

1. It is not relevant to specify what the capital city of Benin is (see line 113); rather, the emphasis should be on what the child survival rate in Benin is. Is EIBF an issue in Benin? These are useful to justify why Benin is an important study context.

Response: This statement has been added “Infant mortality rate has witnessed a marginal decline from 64 to 55 deaths per 1,000 live births from in the last decade.” See lines 123-124

2.  A detailed description of your independent variables is required. Perhaps you can construct a table with the following required information: variables, question asked, response options, and coding strategy.

Response: authors have given a detailed description of the independent variables. See lines 152-171

3.  It is not relevant to indicate which Stata commands were used for the analysis (see lines 175 and 178

Response: Thank you this has been removed

Results
1. Authors are encouraged to interpret the descriptive results and not just report the percentages. For example, the authors report this: “The results showed that 28.9% of the respondents were aged 25-29; 65.8% had no formal education; partners of 54.5% had no formal education whilst 83.3% of the participants were working.”

This can be written as follows: A large share of the respondents (70%) were between the ages of 20 and 34. More than 4 out of 5 of them (83%) were employed; however, most of the respondents (65%) had no formal education. The share of spouses without formal education is also quite high (54%).

Response: Thank you this has been improved. See lines 209-222

2. The authors estimated 3 separate models but only interpreted Model 3. It is surprising why they did not interpret the remaining 2 models, and I would encourage them to do so, at least briefly. It would also be helpful for the authors to explain why they estimated 3 separate models.
Response: Authors did not interpret the models 1 and 2 because these were not the final models. Hence, there is no need to place emphasis on it but to focus on the final model (3) which is of interest for this study. These models helped with ICC interpretation. Also, studies on EIBF, TIBF and other studies (Birhan, et al., 2022; Teshale, & Tesema, 2021) placed importance on the final model.

3. Some of the results are not statistically significant (e.g., education and partner’s education), but the authors discussed them as though they were. Only variables that are statistically significant should be discussed in detail. The authors are also encouraged to ensure they are reporting the right confidence intervals.

Response: Thanks for the comment. This has been addressed see lines 292-294

Discussion

1. It is not clear what the authors mean by “Early initiation of breastfeeding has been considered as the cost effective .... (Line 254)." Cost effective in what sense? Further clarification is required.

Response: Thank you for the comment. This statement has been added to bring clarity to the sentence “According to literature, EIBF is one of the best investments for increasing human capital, stimulating economic growth, and ensuring that all children have equitable access to opportunity. Hence, breastfeeding has been noted to have at least contributed more than $300 billion to the world economy each year.” See lines 2278-280

2.  On lines 258 to 260, the authors noted that “This prevalence is lower than those reported by existing studies in SSA [32, 33, 34] and Nepal [35]. However, it is lower than what has been reported by other studies in Asia [36, 37, 38]. What prevalence rate has been reported for SSA, Nepal and Asia?

Response: Thank you. This comment has been addressed. See lines 286-289

3.  The authors should focus their discussion on variables that are statistically significant.

Response: Thank you for. This comment has been addressed in lines 292-294

4.  On lines 302, the authors note that “there is the need to institute appropriate interventions for mothers who have undergone caesarean section deliveries to initiate breastfeeding their children within an hour after birth." It would be insightful for the authors to briefly clarify what could be considered an appropriate intervention in this case.

Response: Healthcare facilities can implement a strategy to guarantee that babies delivered through caesarean section are breastfed promptly, either in the operating room or recovery area. Nonetheless, medical personnel in the delivery room should have the utmost authority to restrict early initiation of breastfeeding if the mother’s or baby’s health will be jeopardized. See lines 340-344

5. The explanation for the surprising finding that women who were attended to by skilled health professionals were less likely to initiate early breastfeeding (see lines 308 to 310) is not convincing enough and requires further elaboration and clarification. The authors seem to suggest that women who are attended to by skilled health professionals are more likely to experience complications than women who are not, which is quite the opposite. Moreover, cesarean section should not be considered as a possible explanation since it was included in the analysis as a control variable.

Response: thank you for the observation. Lines have been improved to read “This might be due to residual confounding, as studies have shown that women who had their births attended by skilled health professionals are more likely to initiate early breastfeeding.” see lines 349-351

6. On line 317, the authors note that the study did not consider maternal beliefs. Why is this important to consider?

Response: This belief was not captured in the dataset. We believe that maternal beliefs can influence EIBF. That is, the study did not consider maternal knowledge on the benefits of EIBF. See line 358-359

7.  The authors suggest that health professionals must minimize indications of caesarean delivery, setup systems that will enable skin to-skin and establish practices that enable early initiation of breastfeeding among women who have undergone caesarean section (lines 330 to 333). It is not quite clear what the authors mean, and further clarification and elaboration are require.

Response: Thank you for the observation. This statement has been removed.
